# Minutes of the Central New York Homœopathic Medical Society

**Published:** 1884-06

**Authors:** 


### THE MINUTES OF THE CENTRAL NEW YORK HOMŒOPATHIC MEDICAL SOCIETY.

Syracuse, N. Y., March 20th, 1884.

The Central New York Homœopathic Medical Society met in the office of Dr. Wm. Hawley.

Present—Drs. Besemer, Hawley, Seward, Emmons, Brewster, and Nash.

The president and vice being absent, Dr. Seward was chosen chairman and Dr. Nash secretary *pro tem.*

Minutes of last meeting were read and approved.

Dr. Hawley read the resignation of the secretary, C. P. Jennings, which was accepted. Dr. Hawley also suggested that the election of a new secretary be postponed until next meeeting. It was postponed.

Dr. Julius Schmidt presented a paper to the Society, entitled "Pathology Applied," which was read by Dr. Hawley. It was received with a vote of thanks and referred to the Publishing Committee, who ordered its publication.

Dr. Nash reported two cases of diphtheria cured, one with Apis, the other with Lycopod.

Dr. Brewster cured a case in which the symptom which guided him to the selection of Apis was alternate sweating and dryness of the skin; also one with the following symptoms: chilliness, violent pains in back and head, wants hot drinks, whines and finds fault with everything. Cured with Lycop.

Dr. Hawley reported a case of shingles (in himself), pains, burning, and itching, worse nights, nine to three o'clock. Cured with Rhus. tox.$^{75m}$ (Fk.).

Dr. Hawley reported, verbally, a case of typhoid fever. Face mahogany colored; temperature, 103; dry, red tongue, with triangular, red tip. Rhus. tox.$^{30}$ in repeated doses brought no improvement, but in the 75m rapidly cured.

Dr. Nash—Would not the case have recovered the same if the 75m had not been given, but the 30th withdrawn simply.

Dr. Hawley—Don't know, but think not.

Dr. Nash had a case of post scarlatinal dropsy, which rapidly recovered on Hell. nig.$^{33m}$, after the low had been given without any apparent improvement.

Dr. Brewster reported a case that had taken Quinine for chills and fever for a long time. Chills came at ten A. M., lasting two or three hours, followed with fever lasting three to six hours, with violent thirst, numbness of the legs, with almost loss of power in them, dullness of left lung, and severe cough; was pronounced as being incurable and as having consumption by old-school physicians. Chills cured, thirst relieved, cough mitigated, and appetite improved by a single dose of Nat. m.$^{2c}$.

Dr. Besemer cured a case of so-called malarial fever, which had been a long time under the care of Dr. Van De Wasker, of this city. She came to his place on the cars bolstered up with pillows. Guided by the time of chills, thirst, headache, fever, and fever blisters upon lips, Nat. m.$^{cc}$ was prescribed and patient cured in one week.

Dr. Hawley presented and asked for advice in the treatment a case of paralysis agitans of the right arm in a lady sixty years of age, also complains of numbness and pain in nape of neck running down the back.

Dr. Seward advised Mercury.

Meeting adjourned.

